# Dynamic changes of CSF sPDGFRβ during ageing and AD progression and associations with CSF ATN biomarkers

**DOI:** 10.1186/s13024-021-00512-w

**Published:** 2022-01-15

**Authors:** Jun Wang, Dong-Yu Fan, Hui-Yun Li, Chen-Yang He, Ying-Ying Shen, Gui-Hua Zeng, Dong-Wan Chen, Xu Yi, Ya-Hui Ma, Jin-Tai Yu, Yan-Jiang Wang

**Affiliations:** 1 Department of Neurology, Daping Hospital, Third Military Medical University, Chongqing, China; 2 Chongqing Key Laboratory of Ageing and Brain Diseases, Chongqing, China; 3 Shigatse Branch, Xinqiao Hospital, Third Military Medical University, Shigatse, China; 4 Department of Neurology, Qingdao Municipal Hospital, Qingdao University, Qingdao, China; 5 Department of Neurology and Institute of Neurology, Huashan Hospital, State Key Laboratory of Medical Neurobiology and MOE Frontiers Center for Brain Science, Shanghai Medical College, Fudan University, Shanghai, China; 6 State Key Laboratory of Trauma, Burn and Combined Injury, Institute of Surgery Research, Daping Hospital, Third Military Medical University, Chongqing, China; 7 Center for Excellence in Brain Science and Intelligence Technology, Chinese Academy of Sciences, Beijing, China

**Keywords:** Alzheimer’s disease, Ageing, Pericyte, sPDGFRβ, Blood-brain barrier, β-Amyloid, Tau phosphorylation

## Abstract

**Background:**

Loss of brain capillary pericyte is involved in the pathologies and cognitive deficits in Alzheimer’s disease (AD). The role of pericyte in early stage of AD pathogenesis remains unclear.

**Methods:**

We investigated the dynamic changes of soluble platelet-derived growth factor receptor β (sPDGFRβ) in cerebrospinal fluid (CSF), a marker of brain pericyte injury, in transition from normal ageing to early AD in a cognitively unimpaired population aged 20 to 90 years. Association between sPDGFRβ and ATN biomarkers were analyzed.

**Results:**

In lifetime, CSF sPDGFRβ continually increased since age of 20 years, followed by the increases of phosphorylated tau-181 (P-tau181) and total tau (T-tau) at the age of 22.2 years and 31.7 years, respectively; CSF Aβ42 began to decline since the age of 39.6 years, indicating Aβ deposition. The natural trajectories of biomarkers suggest that pericyte injury is an early event during transition from normal status to AD, even earlier than Aβ deposition. In AD spectrum, CSF sPDGFRβ was elevated in preclinical stage 2 and participants with suspected non-AD pathophysiologies. Additionally, CSF sPDGFRβ was positively associated with P-tau181 and T-tau independently of Aβ42, and significantly strengthened the effects of Aβ42 on P-tau181, suggesting that pericyte injury accelerates Aβ-mediated tau hyperphosphorylation.

**Conclusions:**

Our results suggest that pericyte injury contributes to AD progression in the early stage in an Aβ-independent pathway. Recovery of pericyte function would be a target for prevention and early intervention of AD.

**Supplementary Information:**

The online version contains supplementary material available at 10.1186/s13024-021-00512-w.

## Background

Alzheimer’s disease (AD) is the most common form of dementia, with ageing as the best-known risk factor. Impaired clearance of β-amyloid (Aβ) is considered as the main cause of sporadic AD, which accounts for 99% of total cases [1]. Previous studies demonstrated that transportation from brain to blood plays an important role in the clearance of brain Aβ [2, 3]. The blood-brain barrier (BBB) is the predominant way for Aβ efflux out of the brain, and crucial for maintaining normal brain function and homeostasis [4]. Increasing evidence suggests that BBB breakdown occurs in normal ageing and contributes to AD pathogenesis in early stage [5–7].

Brain pericytes, embedded within the wall of brain capillaries, are critical in maintaining the structural and functional integrity of BBB and protecting neuronal and cognitive function [8–10]. In vitro and in vivo studies showed that BBB-associated pericytes were able to phagocytose Aβ and pericyte loss contributed to AD pathologies and disease progression [11–13]. Human studies revealed significant pericyte loss in the brain and elevated soluble platelet-derived growth factor receptor β (sPDGFRβ), a well-known marker of pericyte injury [14], in cerebrospinal fluid (CSF) of patients with mild cognitive impairment (MCI) and Alzheimer’s dementia, indicating a role of brain pericytes in AD pathogenesis [9, 15–17]. However, these studies only observed pericyte changes in symptomatic stages. The roles of brain pericyte injury in transition from normal ageing to preclinical AD remain largely unknown. In the present study, we investigated the dynamic changes of CSF sPDGFRβ in the process of normal ageing and preclinical AD, and its correlation with Aβ and tau clearance across BBB.

## Methods

### Study cohorts

#### CADS cohort

Chongqing Ageing & Dementia Study (CADS) is an ongoing cohort study initiated in 2010, which aimed to explore the evolution mechanisms of ageing to AD, to identify biomarkers of early diagnosis and interventional strategies for AD. All participants were the southwest Chinese population and from Daping Hospital.

In the present study, a total of 303 non-demented participants aged 20 years and older were enrolled from CADS cohort. CSF Aβ42, Aβ40, phosphorylated tau-181 (P-tau181) and total tau (T-tau) levels had previously been measured. Among them, 87 subjects aged 40 years and older were enrolled in CSF/plasma cohort, for whom CSF and contemporaneous plasma Aβ42, Aβ40 and T-tau were simultaneously measured. Subjects were excluded if they had: (1) dementia caused by other neurological diseases rather than AD (e.g., vascular dementia, frontotemporal dementia, dementia with Lewy bodies, etc.) (2); cancers (3); severe cardiac, hepatic, or renal insufficiency (4); mental illness (e.g., schizophrenia) (5); unwillingness to participate in the present study.

According to the National Institute of Aging-Alzheimer’s Association (NIA-AA) criteria [18, 19], participants were divided into stage 0, stage 1, stage 2 and SNAP. In brief, stage 0 was defined as normal cognition with A-T-N-, stage 1 was normal cognition with A + T-N-, stage 2 was normal cognition with A + T/N+ (including A + T + N-, A + T-N+, A + T + N+), SNAP was with A-T/N+ (including A-T + N-, A-T-N+, A-T + N+).

#### CABLE cohort

Chinese Alzheimer’s Biomarker and LifestylE (CABLE) study is an ongoing cohort study initiated in 2017 and mainly focusing on AD risk factor and biomarkers in the northern Chinese Han population from Qingdao Municipal Hospital [20]. In the present study, 21 normal control (stage 0), 42 preclinical AD (23 in stage 1 and 19 in stage 2) and 6 SNAP denoted subjects according to NIA-AA criteria were included. The exclusion criteria were the same as those in CADS cohort.

Demographic characteristics were extracted, including age, sex, education levels, *APOE* genotype, Mini-Mental State Examination (MMSE) and Clinical Dementia Rating (CDR) scores, VRFs including hypertension, type 2 diabetes, hyperlipidemia, chronic heart disease, history of stroke, current smoking status. VRF burden was the sum of these factors.

### CSF and plasma sampling and processing

CSF was processed according to guidelines from Alzheimer’s Biomarkers Standardization Initiative (ABSI) [21]. CSF samples were collected in polypropylene tubues by lumbar puncture. Then, centrifuged at 2000 *g* for 10 min at room temperature within 2 h. The supernatant was aliquoted and stored at − 80 °C until use.

Plasma was sampled and processed according to guidelines for standardized operating procedures (SOPs) [22]. Briefly, fasting blood were collected in EDTA tube and gently inverted 5–10 min; Placed in room temperature for half an hour. Then, centrifuged for at 2000 *g* for 10 min at room temperature; the supernatant was aliquoted and stored at − 80 °C until use. Total processing time was no longer than 2 h from “stick-to-freezer”.

### Measurement of AD core biomarkers

According to global measurement standardization from the Alzheimer’s Association Global Biomarkers Consortium [23], CSF Aβ42, Aβ40, P-tau181 and T-tau levels had previously been measured using enzyme-linked immunosorbent assays (ELISA) kits (INNOTEST, Fujirebio, Belgium) according to the manufacturer’s protocol in both CADS and CABLE cohorts. (Supplementary Table 3). Due to the significant influences of pre-analytical and analytical factors on AD biomarker in different laboratories [24, 25], the cutoff values of ATN biomarkers were determined in CADS and CABLE cohorts, respectively. The cutoff values to define abnormal ATN biomarkers were CSF Aβ42 ≤ 930.35 pg/mL (A^+^), CSF P-tau181 > 48.56 pg/mL (T^+^), CSF T-tau > 284.53 pg/mL (N^+^) in CADS cohort, and Aβ42/40 ratio < 0.022 (A+), CSF P-tau181 > 45 pg/mL (T+), CSF T-tau > 236 pg/mL (N+) in CABLE cohort.

In CADS cohort, plasma Aβ42, Aβ40 and T-tau were simultaneously measured using the commercially available single-molecule array (SIMOA) Human Neurology 3-Plex A assay kit (Quanterix, Massachusetts, USA) on-board of the automated SIMOA HD-1 analyzer. ELISA data and SIMOA data were corrected with the mean values of two age- and sex-matched cognitively normal populations from the same sample. CSF to plasma ratios were calculated with the original concentration detected by SIMOA assay.

### CSF sPDGFRβ measurement

CSF sPDGFRβ levels were measured using the human PDGFR beta ELISA kits (Thermo Scientific, Massachusetts, USA) according to the manufacturer’s protocol. CSF sPDGFRβ levels in CADS and CABLE cohorts were adjusted by mean concentrations of calibrators from age-matched stage 0 individuals from the two cohorts.

### Statistics analyses

The data are expressed as the mean ± standard deviation (SD) for numerical variables or as the count (%) for categorical variables unless special illustration. We tested normality of distribution using the Shapiro-Wilk test and visual inspection of Q-Q plot. CSF Aβ40 and Aβ42 concentrations accorded with normal distribution. The values of CSF T-tau, P-tau181 and sPDGFRβ were log10-transformed to obtain normal distribution. We performed following analysis after excluding outliers by box plots to avoid the influence of extreme values. ANCOVA followed by Bonferroni post hoc analyses were used for multiple comparisons of CSF sPDGFRβ levels, age and participant source were used as covariates. Student’s *t* test or Mann-Whitney *U* test was used for comparisons of continuous variables between two groups as applicable, χ^2^ test was used for comparisons of categorical variables. Pearson or spearman correlation analyses were used to examine the correlations between CSF sPDGFRβ and age, Aβ, tau and MMSE score where appropriate.

To establish the trajectories of biomarkers in lifetime, we plotted every variable against age of every individual. Then we used four different curve-fitting models: linear, quadratic, cubic and exponential. The AIC was used to determine which curve-fitting approach conformed best to the data, and the best curves were generated to reflect the average trajectory of each parameter in lifetime. Finally, we transformed data of each parameter into *Z*-scores for normalization to integrate them in one coordinate system. To test the impact of brain pericyte injury on the relationship between the two classical AD hallmarks, we conducted simple moderation analyses (model 1) to test whether the relationship between CSF Aβ42 and P-tau could be moderated by CSF sPDGFRβ while controlling for age. The significance of the moderation was assessed by PROCESS macro for SPSS using 5000 bootstrap samples.

All hypothesis testing was two-sided, and statistical significance was defined as *P* < 0.05. All statistical computations were performed using Graphpad prism version 8.0 and SPSS version 23 (SPSS, Inc., Chicago, USA).

## Results

### Natural trajectories and evolution sequence of biomarkers for AD and pericyte damage in lifetime

**Fig. 1:**
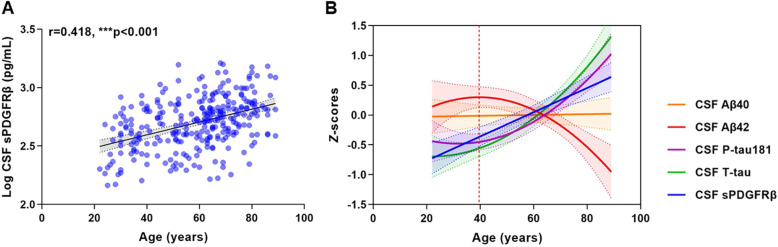
Natural trajectories and evolution sequence of AD core biomarkers and pericyte damage in lifetime. **A** CSF sPDGFRβ levels were progressively increased with age in CADS cohort (*n* = 303). **B** Natural trajectories of AD core biomarkers and sPDGFRβ in CSF. Shading represents 95% CIs. The vertical dashed line at 39.6 years represents the age when CSF Aβ42 began to decline. AD, Alzheimer’s disease; CADS, Chongqing Ageing & Dementia Study; CSF, cerebrospinal fluid; T-tau, total-tau; P-tau, phosphorylated tau; sPDGFRβ, soluble platelet-derived growth factor receptor β

The demographic characteristics of participants were summarised in Table 1. We first investigated CSF sPDGFRβ levels during normal ageing in non-demented subjects aged 20 years and older from CADS cohort. We observed a positive association between CSF sPDGFRβ with age (*r* = 0.418, *p* < 0.001) (Fig. 1A). The correlation between CSF sPDGFRβ and age remained significant after adjusting for vascular risk factor (VRF) burden and *APOE ε4* status (*r* = 0.411, *p* < 0.001), indicating that brain pericyte injury is likely to be an attribute of ageing.

**Table 1:** Characteristics of the study participants

Characteristics	CADS cohort		CABLE cohort
	Total sample	CSF/plasma cohort	
n	303	87	69
Age, years	58.51 ± 16.59	69.60 ± 8.49	66.10 ± 8.02
Gender, female (%)	122 (40.26)	50 (57.47)	32 (46.38)
Education level, years	11.10 ± 3.53	7.38 ± 4.80	8.45 ± 4.14
*APOE ε4* carriers, No. (%)	54 (17.82)	15 (17.24)	11 (15.94)
Hypertension, No. (%)	75 (24.75)	33 (37.93)	35 (50.72)
T2DM, No. (%)	36 (11.88)	19 (21.84)	11 (15.94)
Hyperlipidemia, No. (%)	6 (1.98)	3 (3.45)	2 (2.90)
CHD, No. (%)	13 (4.29)	7 (8.05)	14 (20.29)
History of stroke, No. (%)	5 (1.65)	4 (4.60)	2 (2.90)
Current smokers, No. (%)	75 (24.75)	16 (18.39)	15 (21.74)
CSF sPDGFRβ, pg/mL	556.52 ± 287.93	713.37 ± 285.61	639.39 ± 221.52
CSF Aβ40, pg/mL	11,138.83 ± 4575.39	12,253.49 ± 4481.98	6808.56 ± 3161.62
CSF Aβ42, pg/mL	1314.64 ± 513.43	1329.62 ± 721.32	187.24 ± 115.29
CSF T-tau, pg/mL	182.45 ± 93.06	203.17 ± 67.50	202.83 ± 124.52
CSF P-tau181, pg/mL	43.55 ± 18.46	49.71 ± 20.13	41.12 ± 14.31
CSF/plasma Aβ40	NA	45.88 ± 20.37	NA
CSF/plasma Aβ42	NA	61.01 ± 31.94	NA
CSF/plasma t-tau	NA	30.48 ± 16.07	NA

Data are expressed as the mean ± standard deviation for numerical variables or as the count (%) for categorical variables unless special illustration

*Abbreviations*: *CADS* Chongqing Ageing & Dementia Study, *CABLE* Chinese Alzheimer’s Biomarker and LifestylE study, *CSF* cerebrospinal fluid, *NA* not applicable, *T2DM* type 2 diabetes mellitus, *CHD* chronic heart disease, *T-tau* total tau, *P-tau* phosphorylated tau, *sPDGFRβ*, soluble platelet-derived growth factor receptor β

To investigate the roles of brain pericyte injury in AD progression, we investigated the natural trajectories and evolution sequence of CSF Aβ, tau and sPDGFRβ in lifetime. Individuals with suspected non-AD pathophysiologies (SNAP) were excluded since they are not considered to be in AD continuum. The Akaike information criterion (AIC) showed that linear regression was possibly the best model for fitting the change of Aβ40 and sPDGFRβ over time; whereas a quadratic was better fitting for Aβ42, P-tau181 and T-tau (Supplementary Fig. 1A to E). The integrated graph showed that CSF sPDGFRβ continuously increased since age of 20 years, followed by the increases of P-tau181 and T-tau at age of 22.2 years and 31.7 years, respectively; whereas Aβ42 began to decline since age of 39.6 years, when Aβ may begin to deposit in the brain (Fig. 1B). Our results suggest that pericyte injury occurs earlier than Aβ plaque formation and may participate in AD pathogenesis in the very early stage.

### Dynamic changes of CSF sPDGFRβ in preclinical AD

**Fig. 2:**
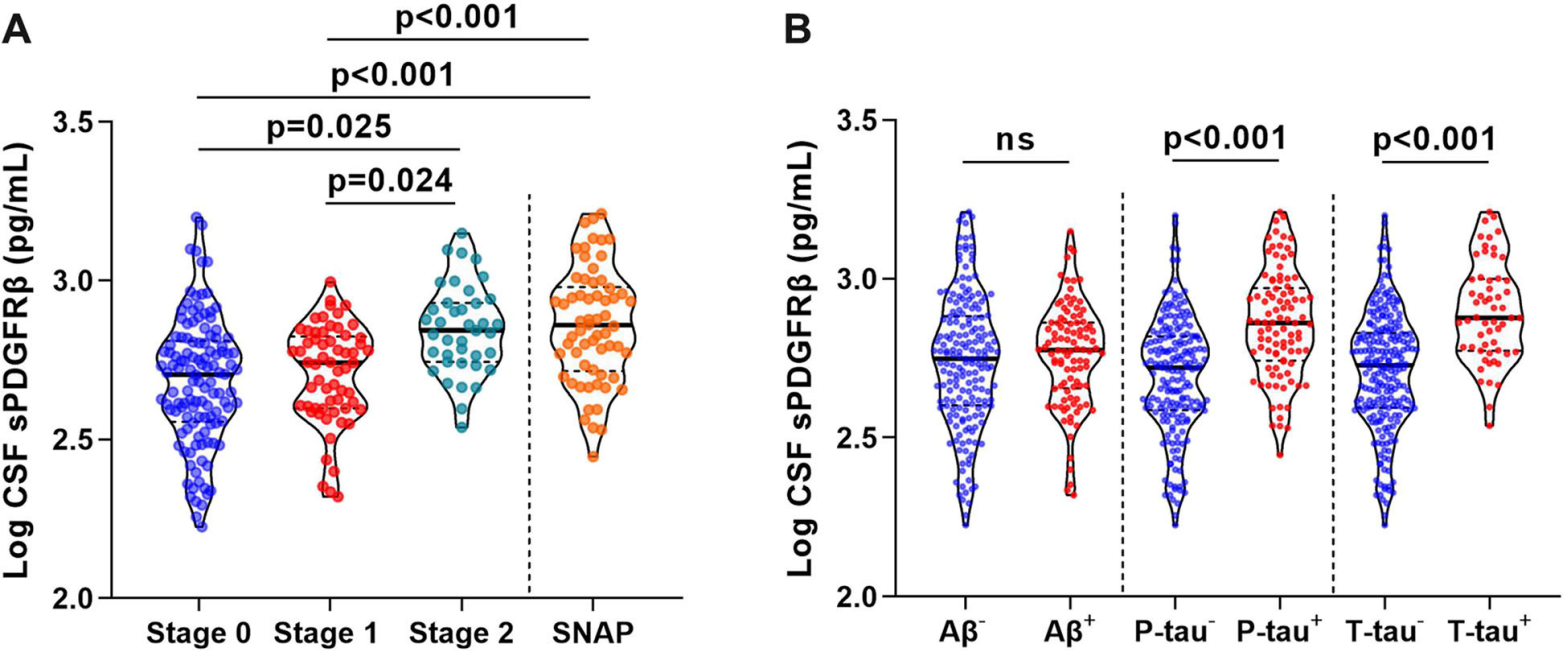
Dynamic changes of CSF sPDGFRβ in transition from normal ageing to preclinical AD. **A** Comparisons of CSF sPDGFRβ among stage 0, stage 1, stage 2 and SNAP patients in CADS and CABLE cohorts. **B** Comparisons of CSF sPDGFRβ based on ATN classification. *P*-values were assessed by ANCOVA followed by Bonferroni corrected post hoc analysis after controlling for age and participant source. AD, Alzheimer’s disease; CADS, Chongqing Ageing & Dementia Study; CABLE, Chinese Alzheimer’s Biomarker and LifestylE study; SNAP, suspected non-AD pathophysiologies; ns, no significance

To investigate the dynamic changes of CSF sPDGFRβ levels in progression from ageing to early AD, we enrolled participants aged 50 years and older at different stages of preclinical AD according to ATN classification system including stage 0, stage 1, stage 2, as well as SNAP, from the CADS cohort and the CABLE cohort (Supplementary Table 1). The results showed that CSF sPDGFRβ was higher in preclinical stage 2 than in normal controls (stage 0) and stage 1 (*p* < 0.05), even after controlling for age and participant source (Fig. 2A). CSF sPDGFRβ didn’t differ by gender, *APOE ε4* status or VRFs (Supplementary Fig. 2A-C).

Additionally, we noted higher CSF sPDGFRβ levels in individuals with SNAP than those in stage 0 and stage 1 after adjusting for age and participant source, which were comparable with those in stage 2 (Fig. 2A), suggesting a closer relationship between pericyte injury with tau hyperphosphorylation and neurodegeneration. Indeed, CSF sPDGFRβ levels increased in P-tau181^+^ and T-tau^+^ individuals, but not in Aβ^+^ individuals (Fig. 2B).

### Associations of CSF sPDGFRβ with tau hyperphosphorylation and neurodegeneration in an Aβ-independent pathway

**Fig. 3:**
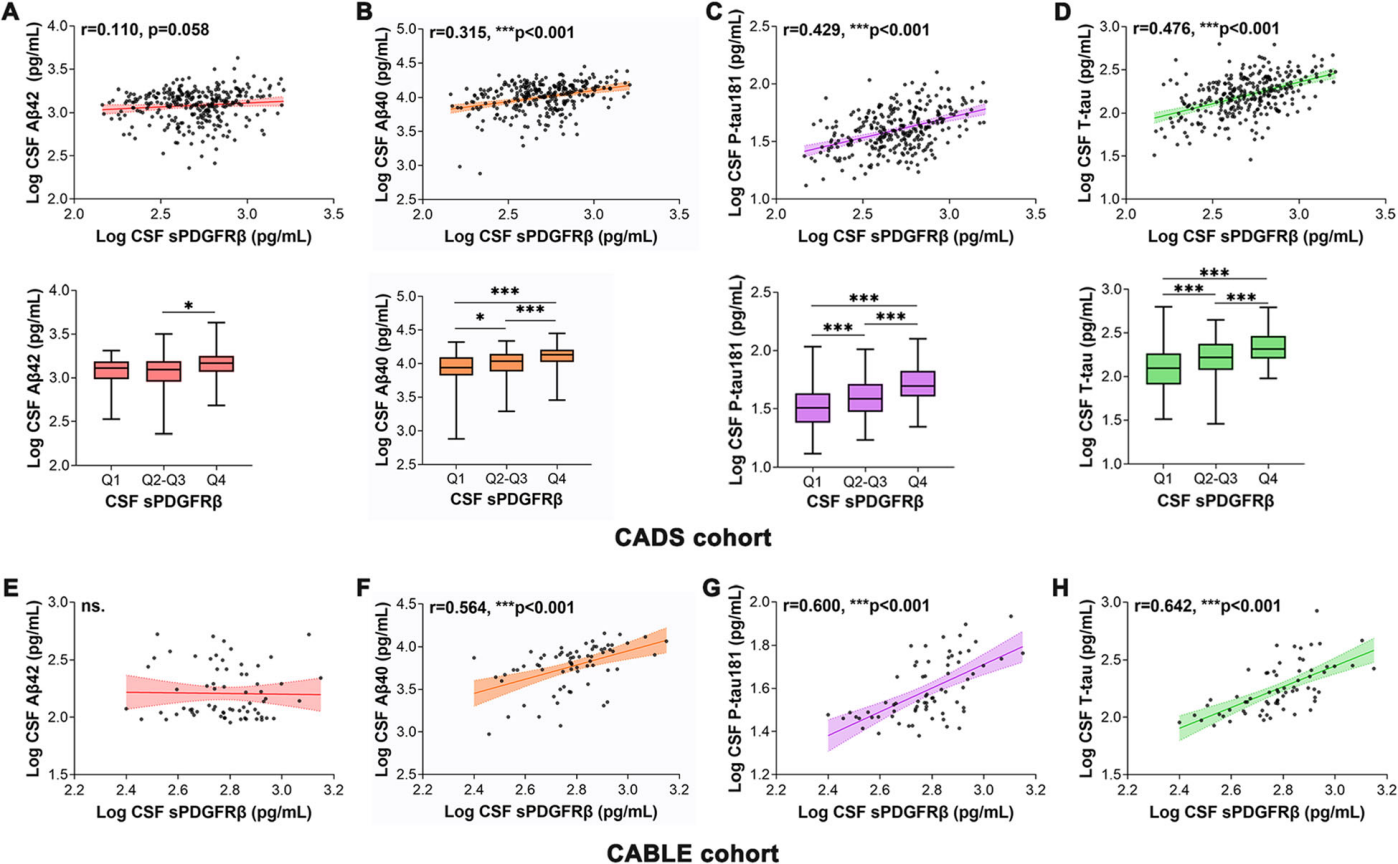
Associations between CSF sPDGFRβ with AD core biomarkers. Relationship between CSF sPDGFRβ and CSF Aβ42, Aβ40, P-tau181 and T-tau in CADS cohort (**A-D**) and CABLE cohort (**E-H**). The best-fit linear regression line is shown and 95% confidence intervals are superimposed. CADS, Chongqing Ageing & Dementia Study; CABLE, Chinese Alzheimer’s Biomarker and LifestylE study. CSF, cerebrospinal fluid; T-tau, total-tau; P-tau, phosphorylated tau; sPDGFRβ, soluble platelet-derived growth factor receptor β; ns., no significance. *p<0.05, *** *p*<0.001

We investigated the associations of sPDGFRβ with AD core biomarkers in CSF. In both CADS and CABLE cohorts, sPDGFRβ was positively correlated with Aβ40, P-tau181 and T-tau even after adjusting for age, gender, *APOE ε4* status and VRF burden. No significant correlation was detected between sPDGFRβ and Aβ42 (Fig. 3A-H, Supplementary Fig. 3). To determine whether the associations between sPDGFRβ with P-tau181 and T-tau are independent of Aβ pathology, we conducted subgroup analysis based on Aβ classification, and found that the correlations between sPDGFRβ with P-tau181 and T-tau remained significant in both Aβ^+^ and Aβ^−^ subgroups (Supplementary Fig. 4). Then we repeated above analyses with CSF Aβ42 and age as covariates, still found positive associations between sPDGFRβ with P-tau181 and T-tau (Supplementary Table 2).

### CSF sPDGFRβ moderated the relationship between Aβ pathology and tau hyperphosphorylation over the course of AD

**Fig. 4:**
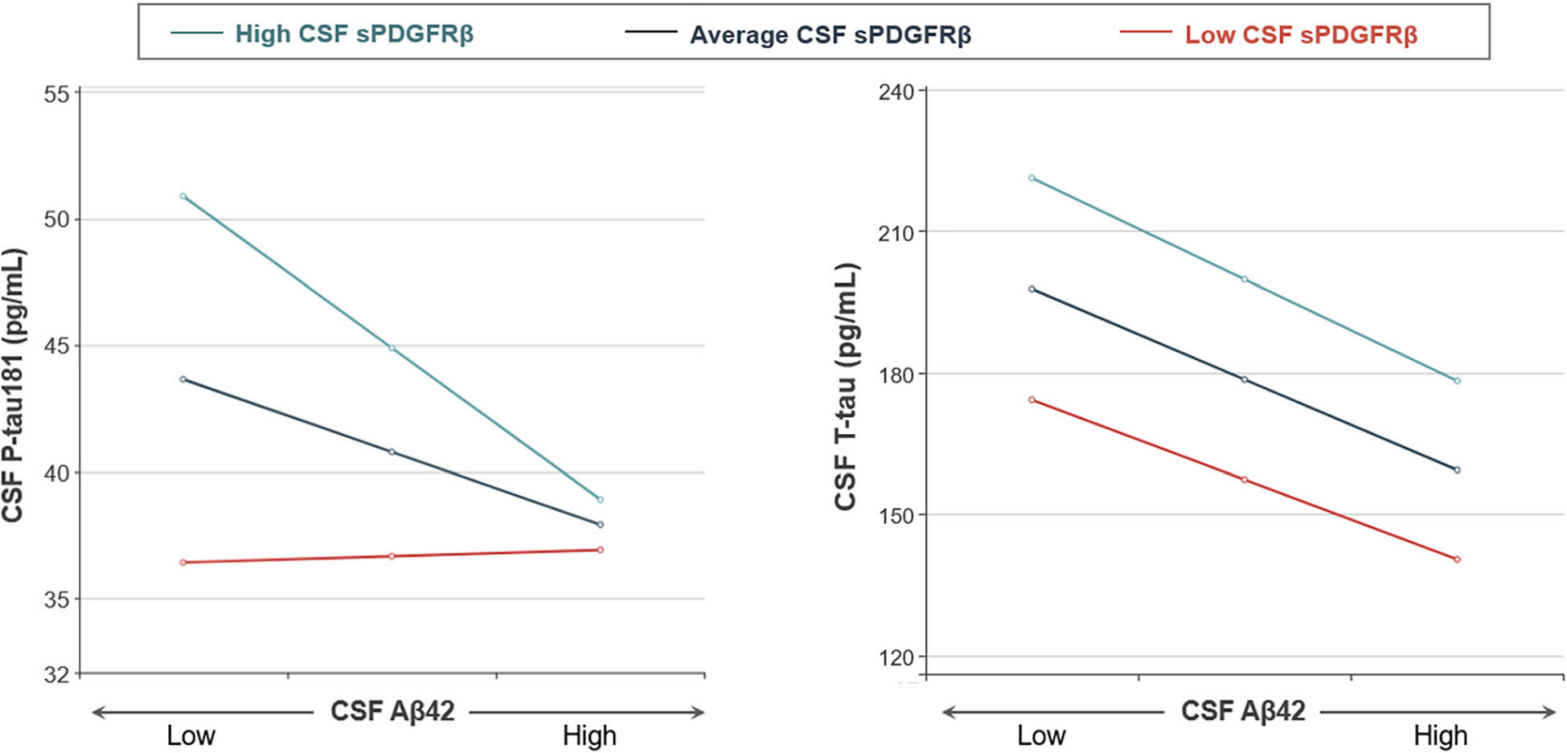
Moderating effects of brain pericyte injury on Aβ-mediated tau hyperphosphorylation and neurodegeneration. **A** CSF sPDGFRβ significantly moderated the effects of CSF Aβ42 on P-tau181. **B** CSF sPDGFRβ didn’t moderate the effects of CSF Aβ42 on T-tau. High, average and low CSF sPDGFRβ represent values of mean - 1SD, mean and mean + 1SD respectively

To further investigate the roles of brain pericyte injury in the relationship of Aβ and tau pathologies over the course of AD, we conducted moderation analyses in individuals aged 50 years and older excluding those with SNAP. The analyses showed that high CSF sPDGFRβ levels significantly strengthened the effect of CSF Aβ42 on P-tau181 with age as a covariate (ΔR^2^ = 0.027, *P* = 0.030). No moderating effect of CSF sPDGFRβ was detected between CSF Aβ42 and T-tau (*P* > 0.05) (Fig. 4).

### CSF sPDGFRβ was associated with CSF/plasma ratios of AD core biomarkers

**Fig. 5:**
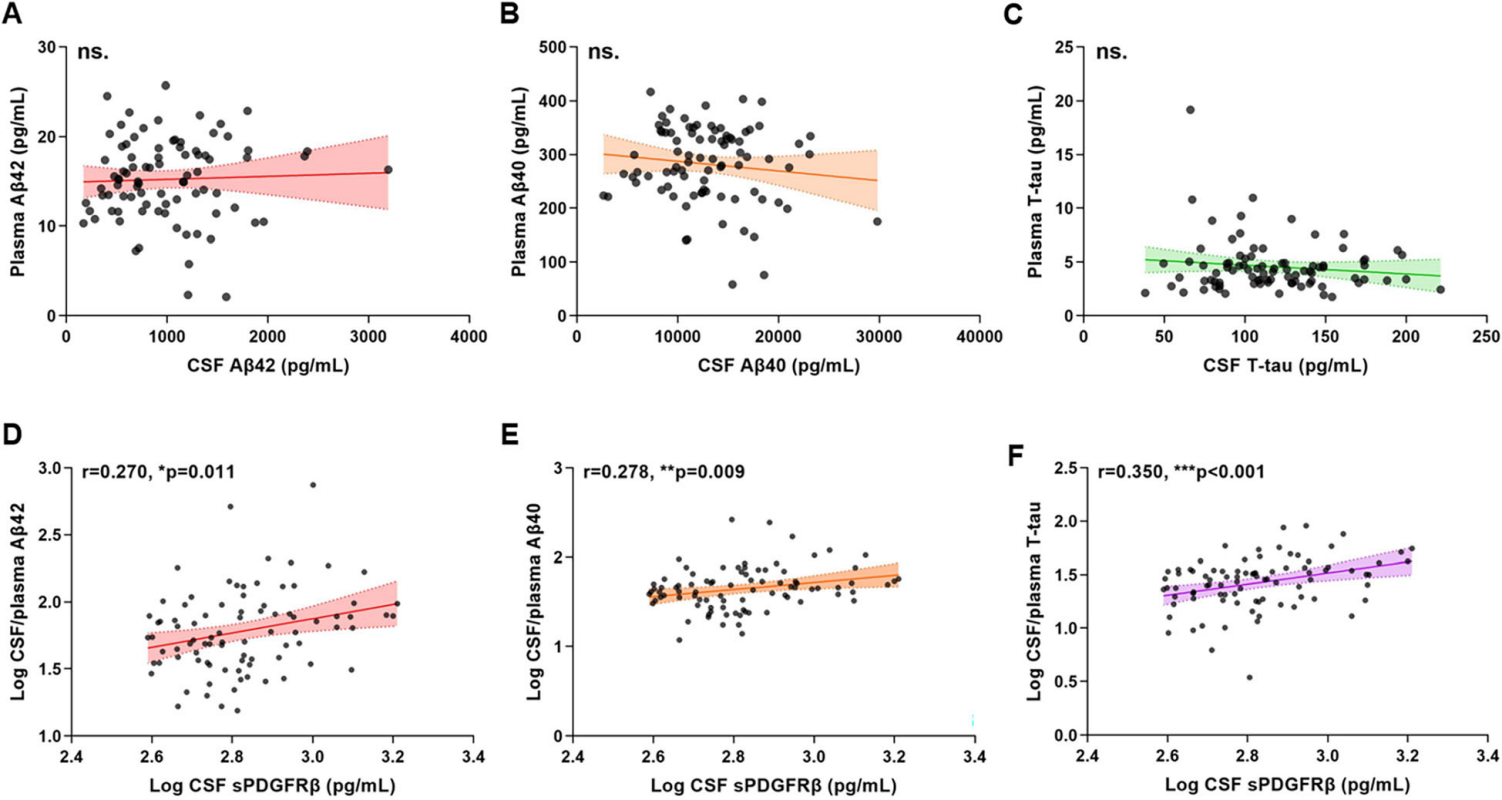
Associations between CSF sPDGFRβ with CSF/plasma ratios of Aβ40, Aβ42 and T-tau in CSF/plasma cohort. **A-C** There were no significant relationships of Aβ40, Aβ42 and T-tau between CSF and plasma. **D-E** CSF sPDGFRβ were positively associated with CSF/plasma ratios of Aβ40, Aβ42 and tTau. The best-fit linear regression line is shown and 95% confidence intervals are superimposed. CSF, cerebrospinal fluid; T-tau, total-tau; sPDGFRβ, soluble platelet-derived growth factor receptor β; ns, no significance. *p<0.05, ** *p*<0.01, *** *p*<0.001

To explore the potential impact of brain pericyte damage on Aβ and tau clearance across BBB, we measured CSF to plasma ratios of Aβ and tau, and examined their associations with CSF sPDGFRβ in CSF/plasma cohort. None of Aβ40, Aβ42 or T-tau in CSF was related to those in plasma **(**Fig. 5A-C**)**. We observed that elevated CSF sPDGFRβ was correlated with higher CSF/plasma ratios of Aβ42, Aβ40 and T-tau, even after adjusting for age, *APOE ε4* status and VRF burden, suggesting Aβ and tau clearance across BBB were impaired (Fig. 5D-E).

## Discussion

Brain pericytes, crucial to communications between the brain and the periphery, has been increasingly linked to ageing and brain disorders, such as cerebral ischemia, white matter diseases, neuroinflammation, etc. [4, 26–28]. Our present study showed an age-dependent increase of CSF sPDGFRβ in living human during normal ageing, independent of VRFs and *APOE ε4* status which were reported to be associated with pericyte-mediated BBB dysfunction [29], suggesting that brain pericyte injury is an attribute of ageing. Previous studies showed massive pericyte loss in the brain and elevated sPDGFRβ levels in CSF of AD patients, implying a role of brain pericytes in AD [9, 15]. Here, we found an elevation of CSF sPDGFRβ in preclinical AD; meanwhile, in the natural trajectories, the increase of CSF sPDGFRβ began earlier than the decline of Aβ42 in CSF, implying that pericyte damage may occur earlier than Aβ deposition, and age-related pericyte damage in brain may contribute to AD pathogenesis in the very early stage of AD. A recent imaging analysis from the Alzheimer’s Disease Neuroimaging Initiative (ADNI) also showed that cerebral vascular dysfunction, as reflected by reduced cerebral blood flow which is regulated by pericytes, occurred earlier than Aβ deposition during transition from healthy control to clinical AD [30]. Taken together, it indicates that age-related pericyte injury contributes to AD progression in the early stage.

Pericyte is the mural cell of brain capillary, the major site for clearing toxic metabolites including Aβ and tau from the brain. Previous studies have reported Aβ phagocytosis roles of brain pericytes, and pericyte loss accelerated Aβ pathology and cognitive decline in AD mice [12, 13]. However, in our present study, we found positive associations between CSF sPDGFRβ with Aβ40, T-tau and P-tau181, but not with Aβ42, which reflects the burden of amyloidosis in brain parenchyma. Consistent with a recent study [17], we did not find correlation between CSF Aβ42 and sPDGFRβ. It is probably because that considerable soluble Aβ42 in the brain deposits into plaque in AD, whereas Aβ40 is predominantly cleared via BBB transporting. Consistently, a previous study reported positive relationships of CSF sPDGFRβ with P-tau181 and T-tau in AD patients and with Aβ42 in cognitively normal controls [15]. Moreover, we also observed positive correlations between CSF sPDGFRβ and CSF to plasma ratios of Aβ42, Aβ40 and T-tau, implying transportation of Aβ and tau across BBB is impaired. The compromised BBB clearance function may partially underlie the poor correlation of these pathological proteins in CSF and blood.

Notably, we found a closer relationship between brain pericyte injury with tau hyperphosphorylation and neurodegeneration independently of CSF Aβ42, indicating that brain pericyte injury may directly contribute to tau pathology and neurodegeneration in an Aβ-independent pathway. One explanation is that considerable soluble extracellular tau proteins in the brain are cleared via BBB transportation; another possibility is that BBB dysfunction permits more blood-derived toxic substances to enter the brain, directly leading to neuronal damage. Besides, as the moderation analyses shown, pericyte injury further accelerates Aβ-mediated tau pathology, thus promotes AD progression. Higher CSF sPDGFRβ has been reported to be linked to cognitive dysfunction and faster cognition decline [29], the close relationship between CSF sPDGFRβ with tau pathology and neurodegeneration provides a theoretical basis for this phenomenon.

**Fig. 6:**
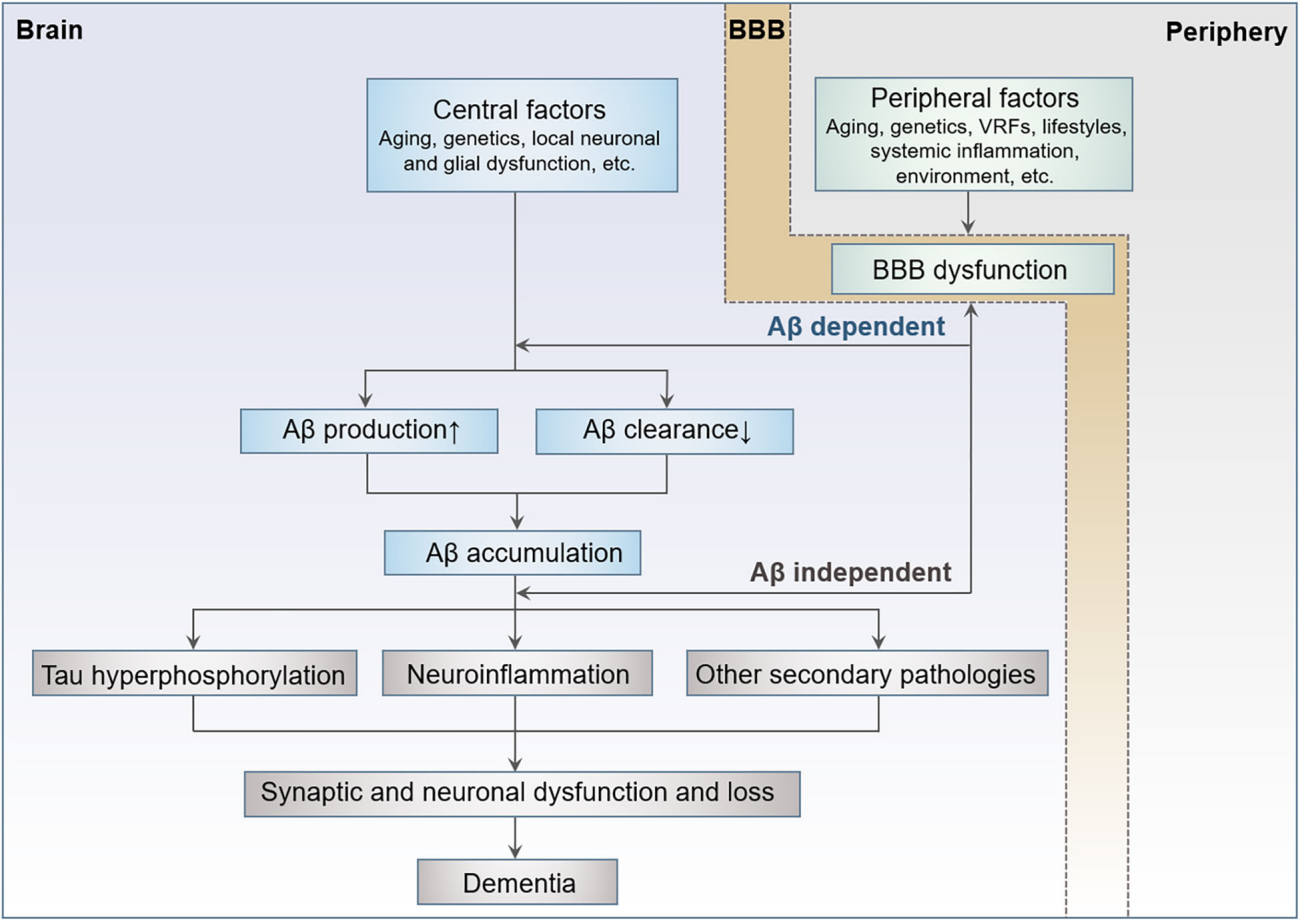
The systemic aetiology hypothesis of AD. Central and peripheral factors synergistically contribute to the development of AD. Central factors initiate Aβ overproduction or insufficient clearance, cause Aβ accumulation and downstream pathologies, eventually lead to neurodegeneration and dementia. Aβ aggregates together with peripheral factors exert damage to BBB. BBB dysfunction leads to neurodegeneration and dementia in both Aβ-dependent and -independent pathways. AD, Alzheimer’s disease; BBB, blood-brain barrier; VRF, vascular risk factor

Cerebrovascular pathologies are common comorbidities in AD patients [31]. Our results suggest that cerebrovascular dysfunction may contribute to AD pathogenesis in both Aβ-dependent and -independent manners. During ageing, cellular senescence together with age-related comorbidities, such as VRFs, systemic inflammation, lifestyles, etc., lead to BBB dysfunction, which further contributes to neurodegeneration in the development of AD **(**Fig. 6**)**. Vascular biomarkers recently have been suggested to be incorporated into the AD research framework—ATX(N) system in AD clinical trials and future clinical practice, where X represents novel candidate biomarkers for additional pathophysiological mechanisms such as neuroimmune dysregulation, synaptic dysfunction and BBB alterations including sPDGFRβ [32, 33]. Our study provides supportive evidence for this proposal.

Although this was a cross-sectional study, the lifespan of participants included almost the whole adult stage, which allowed us to study the natural evolution of AD biomarkers in whole life. All participants in our study were diagnosed and classified according to pathological diagnostic criteria. Our study has several limitations. First, the natural trajectories of biomarkers were drawn in non-demented participants, which need to be confirmed in total population including symptomatic AD patients. Second, the sample size was relatively small, and the results need to be verified in larger-scale study. Thirdly, CSF to plasma ratios of Aβ and tau were an indirect index of BBB clearance, which can also be influenced by blood to brain transcytosis. Our study is an observational study, the causal relationship between pericyte injury with Aβ clearance in the brain and AD pathogenesis needs to be further confirmed.

## Conclusion

Our study reveals the early roles of brain pericyte injury in AD pathogenesis in an Aβ-independent pathway. Our findings imply the contributions of BBB to disease pathogenesis in the early stage of AD. More mechanistic studies of pericyte injury on AD pathogenesis are required. Recovery of pericyte-mediated BBB function provides new opportunities for AD prevention and early treatment.

## Supplementary Information

**Additional file 1.**

## Data Availability

All data needed to evaluate the conclusions in the paper are present in the paper and/or the Supplementary Materials. Additional data that support the findings of this study are available from the corresponding author upon request.

## Abbreviations

AD: Alzheimer’s disease
Aβ: β-amyloid
BBB: blood-brain barrier
sPDGFRβ: soluble platelet-derived growth factor receptor β
CSF: cerebrospinal fluid
CADS: Chongqing Ageing & Dementia Study
P-tau181: phosphorylated tau-181
T-tau: total tau
CABLE: Chinese Alzheimer’s Biomarker and LifestylE
MMSE: Mini-Mental State Examination
CDR: Clinical Dementia Rating
ELISA: enzyme-linked immunosorbent assays
SIMOA: single-molecule array
VRF: vascular risk factor
SNAP: suspected non-AD pathophysiologies
AIC: Akaike information criterion
