# Supplementary material for: Dynamic changes of CSF sPDGFRβ during ageing and AD progression and associations with CSF ATN biomarkers
Source: Mol Neurodegener. 2022 Jan 15;17:9. doi: 10.1186/s13024-021-00512-w (PMC8760673; doi:10.1186/s13024-021-00512-w)
Supplement: Supplementary file 1 — Additional file 1. [file 13024_2021_512_MOESM1_ESM.docx]

**Supplementary Information**

**Title: Dynamic changes of CSF sPDGFRβ during ageing and AD progression and associations with CSF ATN biomarkers**

Jun Wang^1, 2, #^, Dong-Yu Fan^1, 2, 3, #^, Hui-Yun Li^1, 2, #^, Chen-Yang He^1, 2^, Ying-Ying Shen^1, 2^, Gui-Hua Zeng^1, 2^, Dong-Wan Chen^1, 2^, Xu Yi^1, 2^, Ya-Hui Ma^4^, Jin-Tai Yu^5,^ *, Yan-Jiang Wang^1, 2, 6, 7,^ *

1 Department of Neurology, Daping Hospital, Third Military Medical University, Chongqing, China.

2 Chongqing Key Laboratory of Ageing and Brain Diseases, Chongqing, China.

3 Shigatse Branch, Xinqiao Hospital, Third Military Medical University, Shigatse, China.

4 Department of Neurology, Qingdao Municipal Hospital, Qingdao University, Qingdao, China.

5 Department of Neurology and Institute of Neurology, Huashan Hospital, State Key Laboratory of Medical Neurobiology and MOE Frontiers Center for Brain Science, Shanghai Medical College, Fudan University, Shanghai, China.

6 State Key Laboratory of Trauma, Burn and Combined Injury, Institute of Surgery Research, Daping Hospital, Third Military Medical University, Chongqing, China.

7 Center for Excellence in Brain Science and Intelligence Technology, Chinese Academy of Sciences, China.

^#^ These authors contributed equally to this work.

* Correspondence: jintai_yu@fudan.edu.cn (J.-T. Y.); [yanjiang_wang@tmmu.edu.cn](mailto:yanjiang_wang@tmmu.edu.cn) (Y.-J. W.)

**Supplementary Figures**


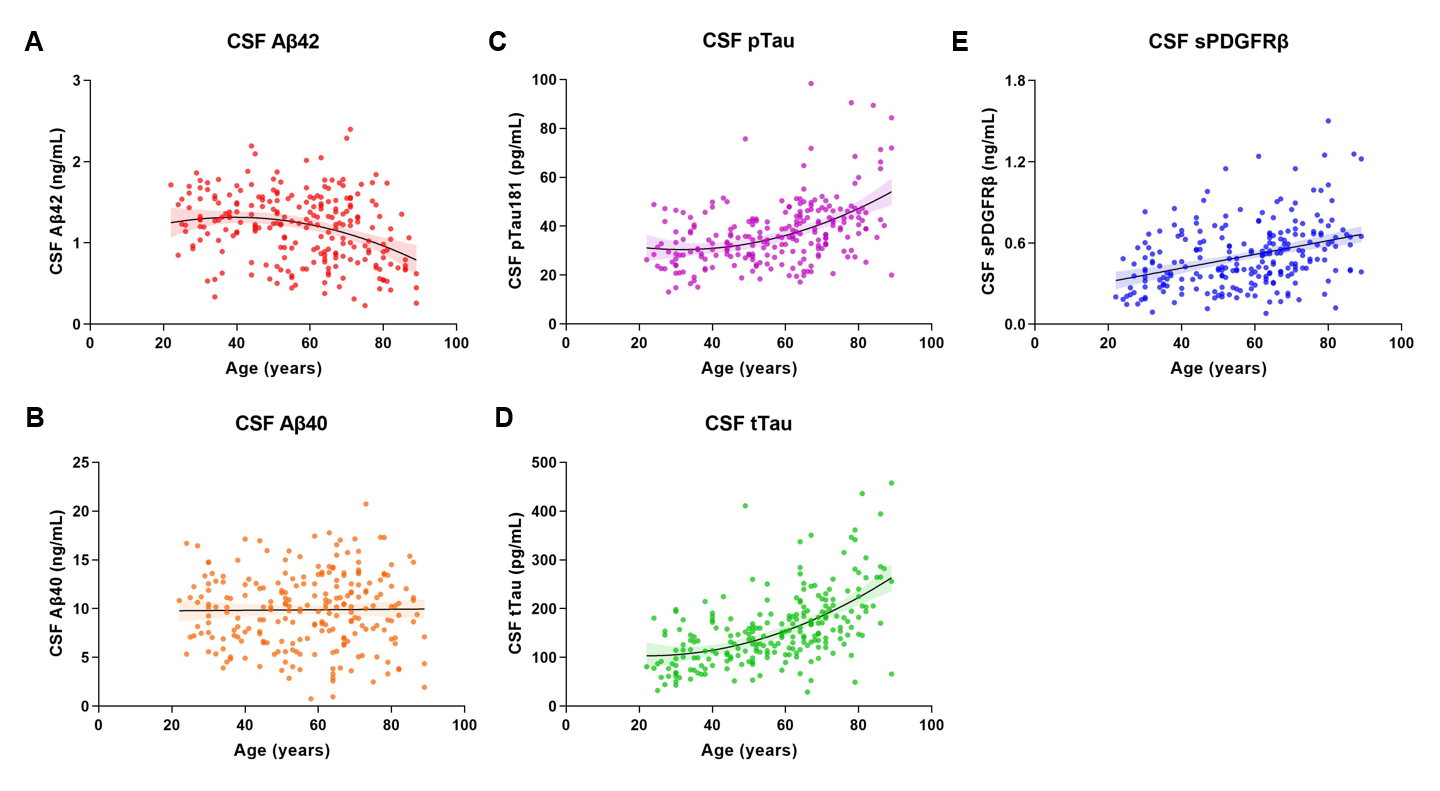


**Supplementary Fig 1. The natural trajectories of CSF Aβ42, Aβ40, P-tau181, T-tau and sPDGFRβ in lifetime.** The best-fit regression line is shown and 95% confidence intervals are superimposed. CSF, cerebrospinal fluid; T-tau, total-tau; P-tau, phosphorylated tau; sPDGFRβ, soluble platelet-derived growth factor receptor β.


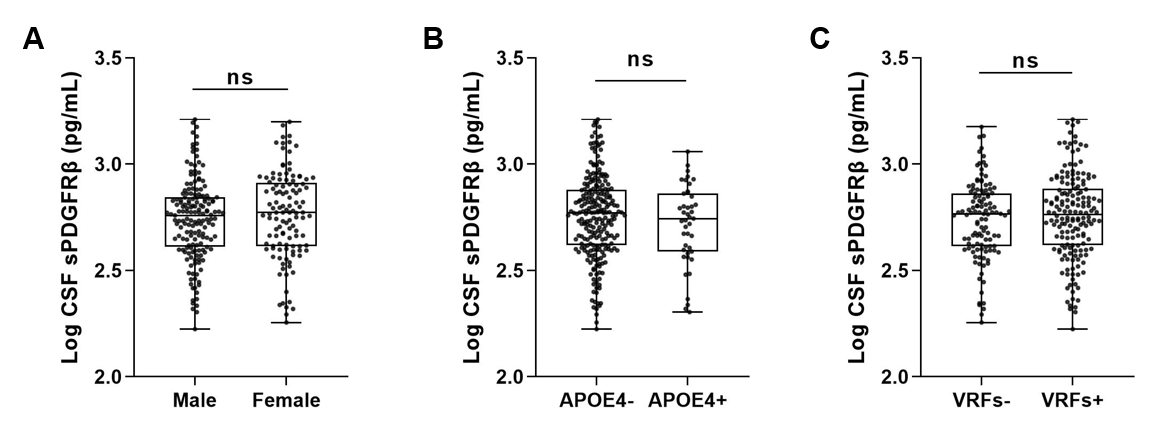


**Supplementary Fig 2. Comparisons of CSF sPDGFRβ levels in gender, VRF status and APOE4 status subgroups in CADS and CABLE cohorts.** CADS, Chongqing Ageing & Dementia Study; CABLE, Chinese Alzheimer’s Biomarker and LifestylE study. CSF, cerebrospinal fluid; sPDGFRβ, soluble platelet-derived growth factor receptor β; VRFs, vascular risk factors; APOE, apolipoprotein E; ns, no significance.


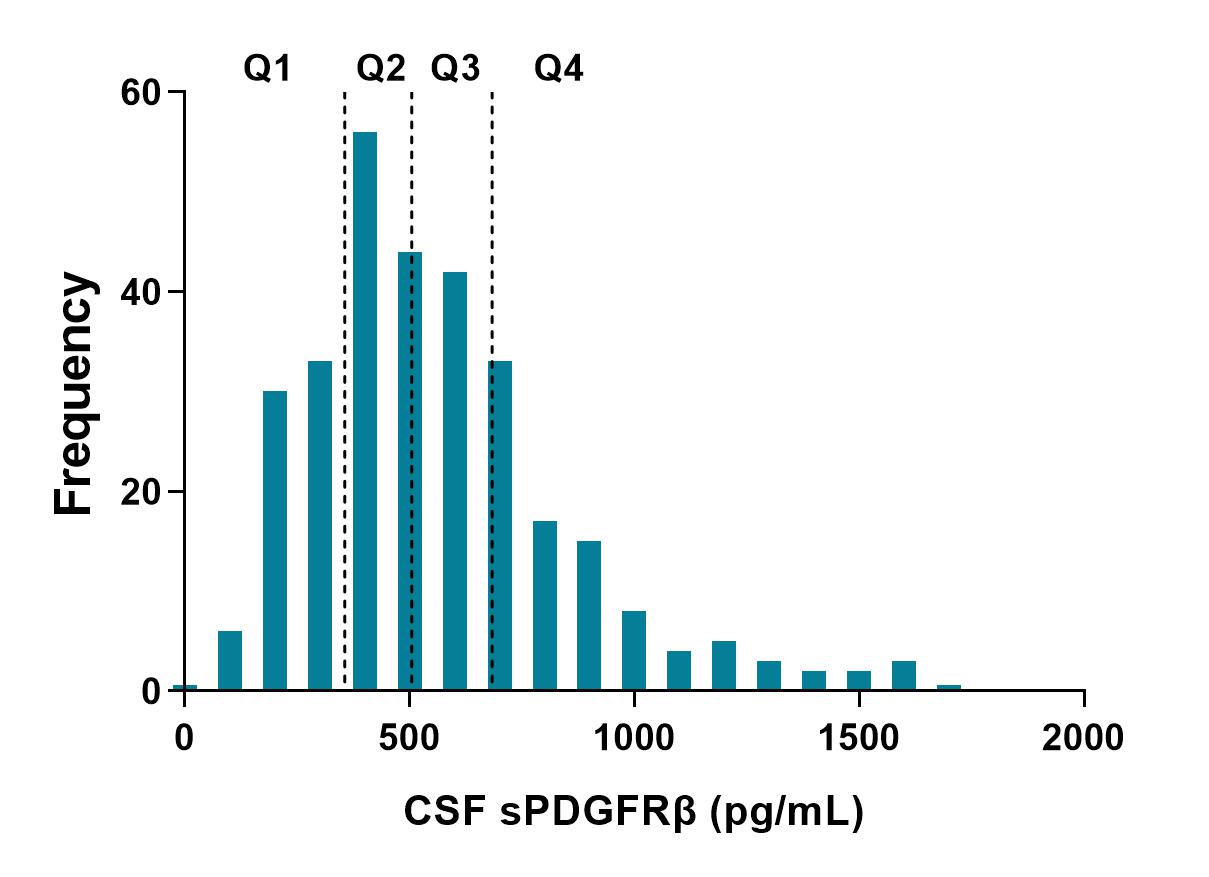


**Supplementary Fig 3. Frequency distribution of CSF sPDGFRβ level in CADS cohort.** CADS, Chongqing Ageing & Dementia Study; CSF, cerebrospinal fluid; sPDGFRβ, soluble platelet-derived growth factor receptor β. **p*＜0.05, ***p*<0.01, *** *p*＜0.001.


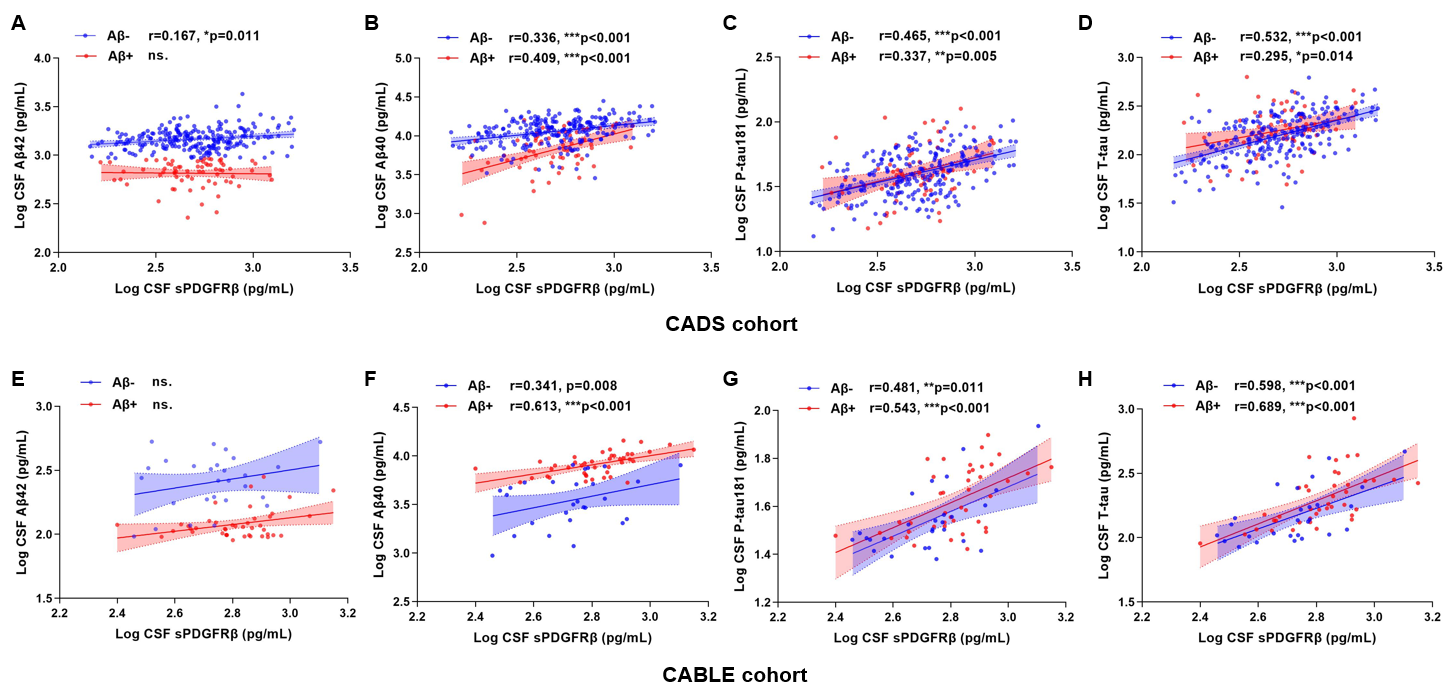


**Supplementary Fig 4. Associations between CSF sPDGFRβ with ATN biomarkers in Aβ^+^ and Aβ^-^ subgroups.** Relationship between CSF sPDGFRβ and CSF Aβ42, Aβ40, pTau181 and tTau in CADS cohort (A-D) and CABLE cohort (E-F). The best-fit linear regression line is shown and 95% confidence intervals are superimposed. CADS, Chongqing Ageing & Dementia Study; CABLE, Chinese Alzheimer’s Biomarker and LifestylE study. CSF, cerebrospinal fluid; T-tau, total-tau; P-tau, phosphorylated tau; sPDGFRβ, soluble platelet-derived growth factor receptor β; ns., no significance. **p*＜0.05, ***p*<0.01, *** *p*＜0.001.

**Supplementary Tables.**

**Supplementary Table 1. Characteristics of participants aged 50 years and older by ATN classification in CADS and CABLE cohort.**

|  | Stage 0 | Stage 1 | Stage 2 | SNAP | P value |
| --- | --- | --- | --- | --- | --- |
| n | 122 | 61 | 39 | 62 |  |
| Age, years | 63.96±9.06 | 67.38±8.81 | 73.00±10.08 | 68.05±8.79 | **<0.001** |
| Female:Male | 54:68 | 21:40 | 15:24 | 27:35 | 0.596 |
| APOE ε4 carriers, No. (%) | 13 (10.7%) | 10 (16.4%) | 12 (30.8%) | 8 (12.9%) | **0.022** |
| Hypertension, No. (%) | 40 (32.8%) | 24 (39.3%) | 22 (56.4%) | 18 (29.0%) | **0.028** |
| T2DM, No. (%) | 17 (13.9%) | 14 (23.0%) | 7 (17.9%) | 9 (14.5%) | 0.448 |
| Hyperlipidemia, No. (%) | 3 (2.5%) | 1 (1.6%) | 1 (2.6%) | 3 (4.8%) | 0.729 |
| CHD, No. (%) | 9 (7.4%) | 8 (13.1%) | 6 (15.4%) | 4 (6.5%) | 0.283 |
| History of stroke, No. (%) | 2 (1.6%) | 3 (4.9%) | 2 (5.1%) | 0 (0%) | 0.204 |
| Current smokers, No. (%) | 31 (25.4%) | 10 (16.4%) | 10 (25.6%) | 19 (30.6%) | 0.321 |
| CSF sPDGFRβ, pg/mL | 534.23±257.64 | 538.99±175.00 | 738.45±244.18 | 781.29±324.93 | **<0.001** |

Categorical variables are presented as numbers and percentages; continuous variables are presented as mean ± SD.

**Abbreviations:** CABLE, Chinese Alzheimer’s Biomarker and LifestylE; AD, Alzheimer’s disease; SNAP, suspected non-AD pathology; HC, healthy control; T2DM, type 2 diabetes mellitus; CHD, chronic heart disease; sPDGFRβ, soluble platelet-derived growth factor receptor β; T-tau, total-tau; P-tau, phosphorylated tau.

**Supplementary Table 2. Association of CSF sPDGFRβ and AD core biomarkers.**

| **CSF biomarker** | **Unadjusted** | |  | **Model 1** | |  | | **Model 2** | |
| --- | --- | --- | --- | --- | --- | --- | --- | --- | --- |
|  | **r** | **P value** |  | **r** | **P value** | |  | **r** | **P value** |
| **CADS cohort** | | | | | | | | | |
| CSF Aβ42 | 0.110 | 0.058 |  | 0.176 | **0.002** | |  | **-** | **-** |
| CSF Aβ40 | 0.315 | **<0.001** |  | 0.330 | **<0.001** | |  | **-** | **-** |
| CSF P-tau181 | 0.429 | **<0.001** |  | 0.324 | **<0.001** | |  | **0.275** | **<0.001** |
| CSF T-tau | 0.476 | **<0.001** |  | 0.346 | **<0.001** | |  | **0.314** | **<0.001** |
| **CABLE cohort** | | | | | | | | | |
| CSF Aβ42 | -0.066 | 0.590 |  | 0.110 | 0.385 | |  | **-** | **-** |
| CSF Aβ40 | 0.564 | **<0.001** |  | 0.429 | **<0.001** | |  | **-** | **-** |
| CSF P-tau181 | 0.600 | **<0.001** |  | 0.560 | **<0.001** | |  | **0.557** | **<0.001** |
| CSF T-tau | 0.642 | **<0.001** |  | 0.620 | **<0.001** | |  | **0.605** | **<0.001** |

Model 1: Adjusted for age, gender, *APOE4* status, and VRF burden.

Model 2: Adjusted for age and CSF Aβ42.

**Abbreviations:** AD, Alzheimer's disease; CSF, cerebrospinal fluid; CADS, Chongqing Ageing & Dementia Study; CABLE, Chinese Alzheimer’s Biomarker and LifestylE; Aβ, Amyloid-β; P-tau, phosphorylated tau; T-tau, total Tau; sPDGFRβ, soluble platelet-derived growth factor receptor β.

**Supplementary Table 3. ELISA kits used in this study.**

| **Biomarker** | **ELISA kits** | **Brand** | **Catalog number** | **Sample Dilution** |
| --- | --- | --- | --- | --- |
| CSF Aβ42 | INNOTEST® β-AMYLOID(1-42) | INNOTEST, Fujirebio, Belgium | 81583 | Without dilution. |
| CSF Aβ40 | INNOTEST® β-AMYLOID(1-40) | INNOTEST, Fujirebio, Belgium | 81585 | 100 fold |
| CSF P-tau181 | INNOTEST® PHOSPHO-TAU (181P) | INNOTEST, Fujirebio, Belgium | 81581 | Without dilution. |
| CSF T-tau | INNOTEST® hTAU Ag | INNOTEST, Fujirebio, Belgium | 81579 | Without dilution. |
| Plasma Aβ40、Aβ40 & T-tau | Human Neurology 3-Plex A assay kit | Quanterix, Massachusetts, USA | 101995 | Without dilution. |
| CSF sPDGFRβ | human PDGFR beta ELISA kits | Thermo scientific, Massachusetts, USA | A111620 | Without dilution. |
